# Biocontrol of fungal phytopathogens by *Bacillus pumilus*

**DOI:** 10.3389/fmicb.2023.1194606

**Published:** 2023-07-25

**Authors:** Jakub Dobrzyński, Zuzanna Jakubowska, Iryna Kulkova, Paweł Kowalczyk, Karol Kramkowski

**Affiliations:** ^1^Institute of Technology and Life Sciences—National Research Institute, Raszyn, Poland; ^2^Department of Animal Nutrition, The Kielanowski Institute of Animal Physiology and Nutrition, Polish Academy of Sciences, Jabłonna, Poland; ^3^Department of Physical Chemistry, Medical University of Białystok, Białystok, Poland

**Keywords:** biological control, eco-friendly agent, spore-forming bacteria, Bacillus, pathogenic organisms

## Abstract

Plant growth-promoting bacteria are one of the most interesting methods of controlling fungal phytopathogens. These bacteria can participate in biocontrol via a variety of mechanisms including lipopeptide production, hydrolytic enzymes (e.g., chitinase, cellulases, glucanase) production, microbial volatile organic compounds (mVOCs) production, and induced systemic resistance (ISR) triggering. Among the bacterial genera most frequently studied in this aspect are *Bacillus* spp. including *Bacillus pumilus*. Due to the range of biocontrol traits, *B. pumilus* is one of the most interesting members of *Bacillus* spp. that can be used in the biocontrol of fungal phytopathogens. So far, a number of *B. pumilus* strains that exhibit biocontrol properties against fungal phytopathogens have been described, e.g., *B. pumilus* HR10, PTB180, *B. pumilus* SS-10.7, *B. pumilus* MCB-7, *B. pumilus* INR7, *B. pumilus* SE52, SE34, SE49, *B. pumilus* RST25, *B. pumilus* JK-SX001, and *B. pumilus* KUDC1732. *B. pumilus* strains are capable of suppressing phytopathogens such as *Arthrobotrys conoides*, *Fusarium solani*, *Fusarium oxysporum*, *Sclerotinia sclerotiorum*, *Rhizoctonia solani*, and *Fagopyrum esculentum*. Importantly, *B. pumilus* can promote plant growth regardless of whether it alters the native microbiota or not. However, in order to increase its efficacy, research is still needed to clarify the relationship between the native microbiota and *B. pumilus*. Despite that, it can already be concluded that *B. pumilus* strains are good candidates to be environmentally friendly and commercially effective biocontrol agents.

## 1. Introduction

Plant diseases cause enormous losses in agricultural production through decreasing yields and the quality of crops, resulting in great economic losses (Rashad and Moussa, 2020). Globally, losses due to plant diseases are estimated to more than 15% for unprotected crops (Chatterjee et al., 2016; Asad, 2022). Importantly, more than 70% of these diseases are caused by fungal phytopathogens (Liu et al., 2017). The most widespread fungal phytopathogens belong to genera such as *Alternaria*, *Aspergillus*, *Botrytis*, *Cladosporium*, *Verticillium*, *Pythium*, *Fusarium*, and *Rhizoctonia* (Djonovic et al., 2007; Zhang et al., 2016; Doehlemann et al., 2017; Krylov et al., 2018; Li and Chen, 2019; Tyśkiewicz et al., 2022). Over the last few decades, chemical fungicides have been the most popular solution for controlling phytopathogens, with recent annual use exceeding one million tonnes per year. However, ongoing climate change and agricultural pollution are boosting the development of eco-friendly agricultural products (Wierzchowski et al., 2021; Zielewicz et al., 2021; Heyi et al., 2022; Wróbel et al., 2022), including safe plant protection products (Mhatre et al., 2018; Köhl et al., 2019; Khan et al., 2022; Lahlali et al., 2022). Environmentally friendly methods of reducing plant phytopathogens include biological control with beneficial bacterial strains (Durán et al., 2018; Pascale et al., 2020). Bacteria are the most ubiquitous known organisms found in the environment and are not evenly distributed. For example, the density of bacteria around the roots of plants is usually considerably higher than the density found in the soil as a whole, which is influenced by root exudates containing, among other things, sugars, amino acids, or organic acids that provide a source of energy for bacteria (Carvalhais et al., 2015; Ojuederie et al., 2019; Basu et al., 2021; Khan et al., 2021; Bhat et al., 2023). Importantly, many bacterial strains are found in or around rhizosphere soil and have the ability to enhance plant growth, hence they are called plant growth-promoting bacteria (PGPB). Besides, PGPB also include endophytic bacteria (Morales-Cedeño et al., 2021). PGPB are capable of promoting plant growth either directly or indirectly. Direct plant growth promotion traits include, e.g., atmospheric nitrogen fixation, indolyl-3-acetic acid (IAA) production, cytokinins, gibberellins production, 1-aminocyclopropane-1-carboxylic acid deaminase (ACC), and phosphorus solubilization (Prakash and Arora, 2019; Dobrzyński et al., 2022a; Ferrusquía-Jiménez et al., 2022; Kaur et al., 2022; Miljaković et al., 2022; Sarmiento-López et al., 2022). Meanwhile, biocontrol traits are described as an indirect mechanism of plant growth enhancement, e.g., production of antibiotics including cyclic lipopeptides, chitinase and glucanase, and triggering of induced systemic resistance (ISR) (Shahid et al., 2021; Dobrzyński et al., 2022a; Mirskaya et al., 2022). The solutions above are in agreement with the contemporary trends introduced by the strategic programs of the EU and are in line with the principles of the European Green Deal (EGD) and the EU Biodiversity Strategy for 2030, which highlight the importance and necessity of agricultural biologicalization and agroecology development, and an increase in the area of ecological crops (Montanarella and Panagos, 2021).

In terms of agricultural application, one of the most interesting bacteria are members of the genus *Bacillus*. It includes mainly Gram-positive, spore-forming bacteria which have a wide distribution in many environments including soil (Nicholson, 2002; Pudova et al., 2022; Yakovleva et al., 2022). Importantly, *Bacillus* spp. strains show significant resistance to environmental stresses, for instance drought, irradiation, UV light, and low nutrient availability–which also increases their potential to enhance plant growth (Nicholson et al., 2000). So far, many species of the genus *Bacillus* have been described as PGPB, e.g., *B. subtilis* (Siahmoshteh et al., 2018), *B. licheniformis* (Gomaa, 2012), *B. amyloliquefaciens* (Siahmoshteh et al., 2018), *B. megaterium* (Mannaa and Kim, 2018), *B. cereu*s (Chauhan et al., 2016), *B. thuringiensis* (Raddadi et al., 2007; Gomaa, 2012), *B. laterosporus* (Sun et al., 2021), *B. vallismortis* (Castaldi et al., 2021), *B. badius* (Zhu et al., 2021), *B. velezensis* (Azabou et al., 2020), *B. endophyticus* (Chauhan et al., 2016; Zhang et al., 2019). In terms of biocontrol agents, the best studied species of *Bacillus* spp. is *B. subtilis*. Due to its properties, including ISR induction, and the lipopeptides or hydrolytic enzymes production, it has already been commercialized as a fungal phytopathogen control agent and currently, there are many preparations on the market containing it in the formulation (Hashem et al., 2019; Samaras et al., 2021). Nevertheless, according to the literature, *B. pumilus* is not inferior in terms of biocontrol; for instance, *B. pumilus* strains can also trigger ISR and produce numerous lipopeptides and enzymes involved in the suppression of fungal phytopathogens. It suggests that the species has a great potential for more frequent commercialization (Jeong et al., 2014). However, before this can happen, it is necessary to look a little deeper into the effectiveness of potential PGPB. Hence, this review aims to gather and summarize the information on the potential of *B. pumilus* in biocontrol and to indicate research gaps that should be filled to increase the effectiveness of *B. pumilus* as a biocontrol agent and contribute to increased interest in commercialization.

## 2. *Bacillus pumilus* biofilm formation

Biofilm is a form of bacterial community that is considered to be the most widespread form of bacterial life in the natural environment (it also occurs in artificial environments, e.g., thrives on plastic). Bacterial cells in biofilms have a higher degree of organization and therefore more advantages than single cells. In a biofilm, bacterial cells form multicellular aggregations encapsulated in a matrix that generally consists of exopolysaccharides (EPS) and fiber proteins as well as extracellular DNA (eDNA) (Sutherland, 2001; Flemming and Wingender, 2010; Diehl et al., 2018; Flemming and Wuertz, 2019). According to the occupied surface, biofilms can be divided into submerged (solid-liquid interface), colonic (solid-air interface), or pellicles (liquid-air interface). Submerged and pellicle biofilms differ in terms of access to oxygen and nutrients, and the preferred niche for biofilm formation appears to depend on the bacterial species (Diehl et al., 2018). For example, *B. subtilis* is mainly known for its architecturally complex colonies and formation of wrinkled pellicle, while, e.g., *Pseudomonas aeruginosa* can show either a pellicle or submerged lifestyle (Tjalsma et al., 2000; Gao et al., 2015; Mielich-Süss and Lopez, 2015).

Bacterial biofilm formation is regulated by different genes and activated by different environmental factors. In terms of the mechanism of biofilm formation, one of the best known bacterial species is *Bacillus subtilis*; its mechanism depends mainly on the de-repression of the *epsA-O* and *tapA-sipW-tasA* operons which are responsible for coding enzymes involved in exopolysaccharides (EPS) synthesis and the amyloid fibril protein TasA (Branda et al., 2004, 2006). De-repression occurs when the Spo0A regulator reaches a threshold level of phosphorylation and leads to repression of the *abrB* which acts as a repressor of the previously mentioned operons (*epsA-O* and *tapA-sipW-tasA*). In addition, Spo0A upregulates the expression of *sinI*, which is responsible for the repression of the matrix gene, *sinR* (Fujita et al., 2005; Kearns et al., 2005).

Interestingly, it has been documented that 23 genes (identified by a random transposon insertion mutagenesis) are responsible for the regulation of biofilm formation in *B. cereus* strain AR156, including the *comER* gene which plays a significant role in both biofilm and spore formation, and is thought to be a part of the regulatory pathway responsible for activation of Spo0A (Xu et al., 2014; Huang et al., 2021; Kulkova et al., 2023). It is conceivable that, due to the affinity to *B. subtilis* and *B. cereus*, most of the molecular mechanisms of biofilm formation in *B. pumilus* may be similar to those in the aforementioned species. Nevertheless, there are still no studies helping to understand the process of biofilm formation in *B. pumilus* and therefore there is an urgent need for future studies.

Effective promotion of plant growth is associated with colonization of the rhizosphere (in the case of rhizobacteria) or plant tissue (in the case of endophytes). In turn, the intensity of soil and plant colonization is linked to biofilm formation, which allows better accessibility to nutrients and thus better biocontrol efficiency (Bhattacharyya and Jha, 2012). In contrast, environmental factors that affect biofilm development include pH, temperature, humidity, oxygen, and metal ions in the rhizosphere or plant (Dutta and Podile, 2010; Zhou et al., 2016). In the case of *B. pumilus*, there is a little research on biofilm formation, which could enhance plant growth promotion. Nevertheless, Zhu et al. (2020a) conducted a biofilm study on *B. pumilus* HR10 which has previously been described as a rhizosphere bacterium involved in biocontrol, including the control of plant phytopathogens and supporting in mycorrhiza formation. Namely, the authors observed a noticeable ease in the formation of a stable biofilm structure on the medium surface. The optimum temperature for the *B. pumilus* HR10 biofilm was 37°C, while the optimum pH was 7.0. Additionally, the study found that the biofilm was not very sensitive to an acidic and alkaline environment. Furthermore, it was proven that the majority of polysaccharide components of plant root exudates enhanced biofilm formation by *B. pumilus* HR10, with glucose having the largest stimulating impact. Besides, low concentrations of such elemental ions as sodium, potassium, calcium, iron, and magnesium promoted biofilm formation (Zhu et al., 2020a). Another study on the biofilm in *B. pumilus* was also conducted on strain HR10 (Zhu et al., 2000b). The study compared the properties of the wild-type strain *B. pumilus* with mutants. Among other things, the authors found that the EPS and protein content produced by the mutants was significantly reduced compared to those in the wild-type bacterial strain, and the swarming ability of both types of HR10 strains was positively correlated with biofilm production. Furthermore, an experiment to determine the degree of colonization of the root system of *Pinus massoniana* seedlings proved that the wild strain could colonize and persist, while the biofilm-free mutants showed a poor ability to colonize (Zhu et al., 2020b). Moreover, another strain of *B. pumilus* FAB10 was able to form an abundant biofilm, produce ACC deaminase, and solubilize phosphorus (*in vitro*) (Ansari and Ahmad, 2019). Interestingly, the authors demonstrated the development of the biofilm of the FAB10 strain at various concentrations of NaCl *in vitro*, and then, using electron microscopy, proved that this strain has the ability to colonize wheat roots at various concentrations of NaCl (0 to 250 mM) in a pot experiment (Ansari and Ahmad, 2019).

Moreover, recent studies indicate that the synthesis of lipopeptide antibiotics, especially surfactin and fengycin, is involved in biofilm formation not only by *B. subtilis* but also likely by *B. pumilus*, which may be a key factor for successful colonization of inoculated plant roots (Penha et al., 2020; Zhu et al., 2020b).

## 3. *Bacillus pumilus* triggered induced systemic resistance (ISR)

Plant growth-promoting bacteria is able to modulate the plant immune system for reaction to a wide range of phytopathogens without direct contact with them. This type of action is called induced systemic resistance (ISR). Importantly, ISR is long-term and permanently protects plants. Bacteria exhibiting these traits are considered biocontrol agents (Pieterse et al., 2014; Stringlis et al., 2018). The mechanism of ISR activation by PGPB is still not fully understood. Despite that, a few ISR triggering traits have been proposed, including lipopeptides, volatile organic compounds (VOCs), flagellin, lipopolysaccharides, and siderophores (Chowdhury et al., 2015; Romera et al., 2019; Ayaz et al., 2021; Yu et al., 2022). In plants, ISR is modulated by the jasmonic acid (JA), salicylic acid (SA), and ethylene (ET) pathways (Shoresh et al., 2005; Yu et al., 2022).

Protection due to ISR triggered by the genus *Bacillus* has been recorded in various diseases, e.g., bacterial pathogens, systemic viruses, root-knot nematodes, late blight diseases, leaf-spotting fungal, crown-rotting fungal pathogen, stem-blight fungal pathogen, and blue mold (Choudhary and Johri, 2009; Li and Chen, 2019; Nie et al., 2019). One of the bacteria of the genus *Bacillus* that elicit ISR against fungal phytopathogens are *B. pumilus* strains. For instance, Enebak and Carey (2000) noted a potential elicitation of ISR in *Pinus taeda* by *B. pumilus* strains including INR7, SE52, SE34, and SE49 against a fungal phytopathogen *Cronartium quercuum* which contributes to a disease called fusiform rust. Moreover, ISR triggering by *B. pumilus* can decrease the severity of anthracnose which is caused by another fungus–*Colletotrichum orbiculare*; the experiment was conducted on cucumbers in field trials (Wei et al., 1996). Subsequently, Jeong et al. (2014) studied the commercial *B. pumilus* strain INR7 (Bayer Crop Science) which has the ability to ISR elicitation and is used in plant biological control against *Colletotrichum orbiculare*, *Rhizoctonia solani*, and *Fusarium* spp. Using the Illumina Genome Analyzer IIx, researchers detected 3 gene clusters present in *B. pumilus* INR7 that encode lipopeptide synthetases including surfactin, bacillibactin, and bacilysin, which may be involved in triggering ISR. Besides, *B. pumilus* strain INR7, *B. subtilis* GB03, and *Curtobacterium flaccumfaciens* were tested individually and in a consortium for biological control of various cucumber phytopathogens including *Colletotrichum orbiculare*. In greenhouse trials, *B. pumilus* in the consortium was shown to have a more effective effect in suppressing pathogens compared to the use of *B. pumilus* alone. In field trials, the efficacy of the consortium in eliciting ISR was confirmed–the consortium inhibited anthracnose symptoms (Raupach and Kloepper, 1998). Interestingly, *B. pumilus* KUDC1732 also suppressed gray leaf spot caused by *Stemphylium lycopersici* in peppers (Son et al., 2014).

Furthermore, *B. pumilus* strains are also capable of elicit ISR against bacterial phytopathogens including *Pseudomonas syringae* pv. lachrymans, *Xanthomonas axonopodis* (Jeong et al., 2014; Li et al., 2020), or *Erwinia tracheiphila* (Jeong et al., 2014) and a fungus-like organisms–Oomycetes including *Peronospora tabacina* (Zhang et al., 2004).

In conclusion, it is worth adding that in the case of ISR elicitation by *B. amyloliquefaciens*, FZB42 is probably the main mechanism in controlling phytopathogens (Chowdhury et al., 2015). Therefore, in *B. pumilus* it may be similar but there is still a lack of studies that could confirm this fact.

## 4. Lipopeptides synthesized by *Bacillus pumilus*

Lipopeptide antibiotics are one of the most commonly produced antibiotics by bacteria of the genus *Bacillus* (Saggese et al., 2022). Lipopeptides are low molecular weight biosurfactants, biodegradable, non-toxic, stable, and environmentally friendly (Meena and Kanwar, 2015; Toral et al., 2018). These compounds are synthesized by non-ribosomal peptide synthases (NRPSs) which are encoded by a cluster of genes, for instance: *bmy* (bacillomycin), *bac* (bacilysin), *srf* (surfactin), and *fen* (fengycins) (Luo et al., 2015; Kim et al., 2017; Fazle Rabbee and Baek, 2020). In addition, gene clusters encoding polyketide synthases (PKSs) that are involved in the synthesis of polyketides (PKs), e.g., macrolactin (*mln*), bacillaene (*bae*), and difficidin (*dfn*), have also been detected in *Bacillus* spp.; PKSs exhibit similar properties to lipopeptides (Chen et al., 2007; Fazle Rabbee and Baek, 2020). The cyclic lipopeptides of *Bacillus* spp. are represented by three major families: surfactins, iturins, and fengycins (Penha et al., 2020). For instance, lipopeptides belonging to the surfactin family (e.g., surfactin, bamilocyn, lichenysin, halobacilin, pumilacidin) detected in *B. pumilus* strains are heptapeptides and have antifungal and antimicrobial activities. In addition, they play an important role in biofilm formation and induce systemic immunity in plants (Abbas et al., 2019; Miljaković et al., 2020). Recent studies have shown that surfactin produced by *B. pumilus* strains HR10 and PTB180 may be involved in the prevention growth of the *Rhizoctonia solani* in *Pinus massoniana* seedlings and may inhibit *Botrytis cinerea* mycelial growth and conidia germination on tomato (Zhu et al., 2020b; Bouchard-Rochette et al., 2022). Furthermore, using Matrix-Assisted Laser Desorption–Ionization-Time of Flight Mass Spectrometry (MALDI-TOF MS) analysis of lipopeptides, Labiadh et al. (2021) demonstrated a diverse variety of molecules in *B. pumilus* such as surfactins, pumilacidin, and kurstakin, which exhibited antifungal ability against *Arthrobotrys conoides* and *Fusarium solani*.

Some heptapeptides of the iturin family (e.g., iturin, bacillomycin, mycosubtilin, mixirins, bacillopeptins, mojavensin, subtulene) and decapeptides of the fengycin family (e.g., fengycin, plipastatin, maltacin) have been found in *B. pumilus* strains; they can inhibit a wide range of fungi and some bacteria (Miljaković et al., 2020). For instance, surfactin and fengycin B extracted from the strain *B. pumilus* W-7 can inhibit the growth of *Phytophthora infestans* (oomycete, fungus-like organisms) in potatoes. Importantly, the mechanism of antifungal action of fengycin B is based on direct inhibition of the fungus, while surfactin induces defense responses in potato by enhancing the expression of genes encoding antioxidant enzymes including *pod*, *pal*, and *cat*; it indicates that the two metabolites act in a synergistic way (Wang et al., 2020). Furthermore, antifungal activity against *Sclerotinia sclerotiorum* was also attributed to *B. pumilus* strain YSPMK11 (isolated from the cauliflower endorhizosphere) which produced iturin A and surfactin. In a 2-year experiment under field conditions, *B. pumilus* strain YSPMK11 reduced disease severity caused by *Sclerotinia sclerotiorum* in cauliflower by 93% (Kaushal et al., 2017). Interestingly, isolated from date palm, strain *B. pumilus* showed antagonistic activity against *Rhizoctonia solani*, *Botrytis cinerea R16*, *Galactomyces geotrichum* MUCL 28959, and *Verticillium longisporum* O1, which is associated with the presence of genes encoding lipopeptide synthases of the mycosubtilin and bacillomycin (iturin family) and the pumilacidin (surfactin family), which inhibit fungal growth (El Arbi et al., 2016).

*Bacillus pumilus* is also able to suppress bacterial phytopathogens. For instance, an *in vitro* study by Nikolić et al. (2019) showed that a mixture of *Bacillus pumilus* SS-10.7 and *B. amyloliquefaciens* strains SS-12.6 and SS-38.4 was able to synthesize lipopeptides such as surfactin, fengycin A, and iturin A which inhibited the growth of *Pseudomonas syringae* pv. aptata on sugar beet.

In addition, there are reports that *B. pumilus* strains produce other antibiotic substances including bacilysin (dipeptide antibiotic), tetaine (dipeptide antibiotic), bacircine, amino sugar NTD, bacteriocin (antimicrobial peptide), phenazine (heterocyclic antibiotic), amicoumacin A, paenilamicin, and subtilin, which also exhibit antibacterial and antifungal properties (Sansinenea and Ortiz, 2011; Özcengiz and Ögulur, 2015; Padaria et al., 2016; Toymentseva et al., 2019; Maksimova et al., 2021; Islam et al., 2022; Khatoon et al., 2022).

Finally, it is worth mentioning that most antibiotic substances can be found in relatively low concentrations near the roots colonized by PGBP of the genus *Bacillus*. The exception is surfactin, which has been detected in the root environment in much higher concentrations, accounting for more than 90% of the total lipopeptides. For instance, such lipopeptides as iturin and fengycin have been found in much smaller amounts, and moreover, most of the antibacterial polyketides and other bioactive compounds have so far not been reported at all in the environment of roots colonized by the members of the genus *Bacillus* (Nihorimbere et al., 2012; Debois et al., 2014). Therefore, it is suggested that perhaps surfactin is one of the main substances involved in the suppression of plant pathogens by *Bacillu*s spp. Moreover, it is speculated that antibiotic compounds are not so heavily involved in the direct suppression of phytopathogens, but are more related to ISR elicitation (Chowdhury et al., 2015).

## 5. Hydrolytic enzymes produced by *Bacillus pumilus*

Other biocontrol agents are hydrolytic enzymes including chitinase, cellulase, glucanase, and protease, which break down components of the fungal cell wall (Branda et al., 2004, 2006; Mielich-Süss and Lopez, 2015). Chitinases, cellulases, glucanases, and proteases are enzymes produced by various bacteria such as *Aeromonas*, *Azospirillum*, *Bradyrhizobium*, *Serratia*, *Vibrio*, *Streptomyces*, *Bacillus*, and fungi such as *Aspergillus*, *Fusarium Trichoderma*, and *Penicillium*, plants, Actinobacteria, arthropods, etc. (Juturu and Wu, 2014; Bělonožníková et al., 2022; Díaz-Díaz et al., 2022; Dobrzyński et al., 2022b; Fatima et al., 2022; Gómez-de la Cruz et al., 2022; Yin et al., 2023).

Both *B. pumilus* and related species are able to synthesize the above-mentioned hydrolytic enzymes under various environmental conditions, which makes them potential biocontrol agents (Martínez-Zavala et al., 2020; Dobrzyński et al., 2021; Dimkić et al., 2022). Among hydrolytic enzymes, chitinolytic enzymes are the most important for the biocontrol of fungal phytopathogens. Chitinases are encoded by a number of genes that include, e.g., *chiA*, *chiB*, *chiC and chiD*, *chiF*, *chiG*, *chiR*, *chiW*, and *chiX* (Alam et al., 1996; Hamilton et al., 2014; Danişmazoğlu et al., 2015; Martínez-Zavala et al., 2020; Azizoglu et al., 2021; Kumar et al., 2022). The efficacy of chitinases produced by *B. pumilus* against fungi has been documented in a few studies. For instance, Rishad et al. (2017) reported that *B. pumilus* MCB-7 showed chitinase activity (3.36 U mL^–1^) even at temperatures up to 60°C and high saline concentration. Both crude and purified chitinase exhibited fungistatic activity against important agricultural phytopathogens such as *Fusarium oxysporum*, *Aspergillus flavus*, *Aspergillus fumigatus*, *Aspergillus niger*, and *Ceratorhiza hydrophila* (Rishad et al., 2017). Moreover, based on clear zones on chitin agar, Gurav et al. (2017) isolated the strain *B. pumilus* RST25. The strain exhibited the highest chitinase activity at 96 h (51.7 U mL^–1^) of culture under submerged conditions. Importantly, *in vitro* assay of *B. pumilus* RST25 chitinolytic potential (crude and purified enzyme) showed significant antifungal activity against pathogens such as *F. solani* and *A. niger.* Moreover, after wheat (*Triticum aestivum*) seeds inoculation, the strain RST25 was able to reduce fungal infections (Gurav et al., 2017). Another study also showed that *B. pumilus* (strain JUBCH08) is capable of antagonistic activity against *F. oxysporum* (45% antagonism after dual plate analysis); the highest chitinase activity of *B. pumilus* JUBCH08 was obtained at 35°C after 72 h of submerged fermentation. Interestingly, the chitinase was thermostable and active in alkaline conditions, which indicates the possibility of the use of this strain in biotechnological application (Bhattacharya et al., 2016). Furthermore, Agarwal et al. (2017) conducted a study on biocontrol *Rhizoctonia solani* and *Fusarium oxysporum* using *B. pumilus* MSUA3; the authors observed a significant decrease in the disease index of *Fagopyrum esculentum* after *B. pumilus* MSUA3 inoculation in gnotobiotic conditions.

In terms of other lytic enzymes, *B. pumilus* JK-SX001 is able to produce protease and cellulase (Ren et al., 2013). As evidenced, metabolites of strain JK-SX001 could significantly suppress the growth of three phytopathogens including *Phomopsis macrospora*, *Cytospora chrysosperma*, and *Fusicoccum aesculi*. Importantly, this strain exhibited antagonistic activity against the above-mentioned pathogens on seedlings of polar under greenhouse conditions. Besides, *B. pumilus* JK-SX001 enhanced plant growth by direct stimulation, e.g., improved biomass production and shoot length (Ren et al., 2013). Antifungal activity has also been documented by other authors, as shown in Table 1.

**TABLE 1:** Antifungal activity by *Bacillus pumilus.*

*B. pumilus* strains	Origin	Plant species	Pathogens	Extracellular enzymes					References
				Cellulase	Chitinase	β -1,3-glucanase	β -endoglucanase	Protease	
*B. pumilus* S9	Soil	Rice	Antifungal activity against *Magnaporthe oryzae*	+	−	−	−	+	Sha et al., 2020
*B. pumilus* CV9	Rhizosphere of *Cicer arietinum* L.	–	Antifungal activity against *Alternaria alternata*, *Colletotrichum capsici*, *Fusarium oxysporum* f. sp. ciceri, *Fusarium solani*, *Macrophomina phaseolina*, *Rhizoctonia solani*, *Sclerotium rolfsii*, *Sclerotinia sclerotiorum*	+	+	−	+	+	Sharma et al., 2019
*B. pumilus* SG2	Saline soils	–	Antifungal activity against *Fusarium graminearum* and *Bipolaris sorokiniana*	−	**+**	−	−	−	Shali et al., 2010
*B. pumilus* M3-16	Shallow salt lake in Tunisia	–	Antifungal activity	Untested	Untested	Untested	Untested	+	Essghaier et al., 2009
*B. pumilus* U5	Soil samples collected from various locations in Iran	–	Untested	Untested	+	Untested	Untested	Untested	Tasharrofi et al., 2011
*B. pumilus* SG2	High salty ecosystem (Iran)	–	Antifungal activity *Rhizoctonia solani*, *Verticillium* sp., *Nigrospora* sp., *Stemphylium botryosum*, and *Bipolaris*	Untested	+	Untested	Untested	Untested	Ghasemi et al., 2010

## 6. Microbial volatile organic compounds as fungal growth-limiting agents

Microbial volatile organic compounds (mVOCs) that can be produced by bacteria are also considered a biocontrol agent (Hassan et al., 2017). VOCs are low molecular weight compounds that readily evaporate and diffuse at ordinary temperatures and pressures (Schulz-Bohm et al., 2017; Poveda, 2021). VOCs mainly belong to following groups of chemical compounds: alcohols (e.g., 2-methylbutan-1-ol, ethanol, 2-phenylethanol), esters, aldehydes, alkanes, alkenes, acids, ketones, benzenoids, pyrazines, terpenes, sulfur compounds, and nitrogen compounds (Buzzini et al., 2003; Fialho et al., 2011; Schmidt et al., 2015). Whereas mVOCs that exhibit antifungal activity include for example caryophyllene, dimethyl disulfide, dimethyl trisulfide, hydrogen cyanide, 2-methylpyrazine, S-methyl thioacetate, 2,3,6-trimethylphenol, undecan-2-one, dodecan-2-one, dodecan-2-one, nonan-2-one, decan-2-one, benzonitrile, and acetoin (Arrebola et al., 2010; Schmidt et al., 2015; Tyc et al., 2015; He et al., 2020). Importantly, in most cases bacterial VOCs are found in low concentrations and have a complex composition. Moreover, their concentration and abundance is influenced by many factors, such as the type of medium, culture conditions, and physiological states of the microorganisms (Elkahoui et al., 2015; Tyc et al., 2015). It is also worth adding that the antifungal efficacy of mVOCs-producing bacteria depends on the composition of these compounds and, as a rule, it is a composition of several or more compounds that can effectively inhibit fungal growth (Poveda, 2021).

Microorganisms capable of suppressing fungal growth through mVOCs include representatives of genera such as *Bacillus* spp. (Liu et al., 2008; Arrebola et al., 2010), *Streptomyces* spp. (Wang et al., 2013), *Pseudomonas* spp. (Elkahoui et al., 2015; Rojas-Solís et al., 2018), *Tsukamurella* spp., *Dyella* sp., *Janthinobacterium* spp., *Chryseobacterium* sp. (Tyc et al., 2015), and *Collimonas* spp. (Garbeva et al., 2014). While members of the genus *Bacillus* capable of producing mVOCs include bacteria such as *Bacillus amyloliquefaciens* (Gotor-Vila et al., 2017), *Bacillus subtilis* (Arrebola et al., 2010), and members of related genus *Paenibacillus polymyxa* (Liu et al., 2008).

Bacteria of the genus *Bacillus* are capable of producing compounds that inhibit the growth of fungi such as *Penicillium* sp. (Andersen et al., 1994), *Trichoderma* sp., *Botrytis cinerea* (Gotor-Vila et al., 2017) *Fusarium oxysporum* (Minerdi et al., 2009; Yuan et al., 2012), *Botryosphaeria berengeriana* (Zhang et al., 2010), and *Colletotrichum gloeosporioides* (Lee et al., 2012). However, there are several broad-spectrum antifungal strains against plant fungal diseases. So far, in the case of *B. pumilus*, there have been few studies describing the possibility of mVOCs production (against fungal phytopathogens) by this bacterial species. In this regard, Liu et al. (2008) conducted a study on *B. pumilus* isolated from the rhizosphere of cucumber–*B. pumilus* BSH-4 and *B. pumilus* ZB13. The aforementioned mVOCs-mediated strains inhibited the growth of fungi such as *Sclerotinia sclerotiorum*, *Botrytis cinerea*, *Alternaria brassicae*, *Alternaria solani*, *Ascochyta citrullina*, *Fusarium oxysporum*, *Fusarium graminearum*, *Cercospora kikuchii*, *Rhizoctonia solani*, *Phoma arachidicola*, and *Verticillium dahliae*. In addition, the authors, using GC-MS, showed that the mVOCs produced by either strains of *B. pumilus* include compounds, e.g., 2,4-decadienal, diethyl phthalate, n-hexadecanoic acid, and oleic acid. Interestingly, it was also shown that, compared to *B. subtilis* BL02 and *Paenibacillus polymyxa* BMP-11, either strains of *B. pumilus* produced less volatile substances, which may have been influenced by factors such as the natural dissimilarity of these species of bacteria and the conditions of cultivation including the type of medium and temperature (Liu et al., 2008). Furthermore, Morita et al. (2019) studied the antifungal effect of volatile organic compounds produced by *B. pumilus* TM-R, which was cultured on four different media; its antifungal activity was evaluated against several fungal species including *Fusarium oxysporum*, *Cladosporium cladosporioides*, *Alternaria alternata*, *Curvularia lunata*, *Aspergillus niger*, *Cladosporium cladosporioides*, and *Penicillium italicum*. In dual plate experiment, the bacteria clearly inhibited the growth of five fungi: *Fusarium oxysporum*, *Cladosporium cladosporioides*, *Alternaria alternata*, *Curvularia lunata*, *Cladosporium cladosporioides*, and *Penicillium italicum*, but enhanced the growth of *Aspergillus niger*. In contrast, in tests conducted on a larger volume (12-L medium), the level of antifungal activity was lower, but despite this, the *B pumilus* TM-R cultured on TMEA medium still seeded a high level of growth inhibition of the pathogens tested, especially against *P. italicum* (growth suppression levels reached 93%). Interestingly, the authors detected 32 mVOCs in the tested strain using the GC-MS technique. The content of the compounds and their concentrations were dependent on the culture media. The authors identified dominant mVOCs (that limit fungal growth) including ethanol, S-(−)-2-methylbutylamine, methyl isobutyl ketone, and 5-methyl-2-heptanone (Morita et al., 2019).

Besides, *B. pumilus* in combination with *B. amyloliquefaciens* and *Exiguobacterium acetylicum* has been shown to inhibit the growth of *Peronophythora litchii* (*in vitro*) and disease on litchi fruit (*in vivo*) through the secretion of mVOCs such as α-farnezen, 1- (2-aminophenyl) etanon, benzotiazol (Zheng et al., 2019).

Importantly, as mentioned in an earlier section, studies conducted on other *Bacillus* spp. suggest that mVOCs are involved in ISR induction and may be a major mechanism of phytopathogens control (Chowdhury et al., 2015).

## 7. *Bacillus pumilus* effect on the indigenous microbiota and its post-inoculation monitoring of abundance

As evidenced by research, it is assumed that the structure of the microbial community colonizing plant roots is important for the plant health and resistance to pathogens. Thus, the impact of plant growth-promoting bacteria on the native microbiota and their survival in soil and plant tissues (in the case of endophytes) may be crucial for the efficiency of their application (Manfredini et al., 2021; Kulkova et al., 2023; Wróbel et al., 2023). For instance, after the application against poplar canker, the abundance of endophytic strain, *B. pumilus* JK-SX001 (GFP-labeled strain and evaluated by confocal laser scanning microscopy) has persisted for a long time and its content was higher in roots and stems than in leaves (Huang et al., 2012). Furthermore, *B. pumilus* SQR-N43 was also analyzed using GFP tagging and after 2 weeks from the application of concentration of 10^8^ and 10^9^ cells g^–1^ as well as 10^5^ and 10^6^ cells g^–1^ (study on biocontrol of *Rhizoctonia solani*) the biofilm on the roots was observed. Importantly, colonization of the root by this strain has been recorded in the root apex and in certain regions of the elongation and differentiation zone of plant roots (Huang et al., 2012). Persistence of *B. pumilus* after application was also studied by qPCR technique. Win et al. (2018) showed that after 2 and 5 weeks from inoculation, the population of *B. pumilus* TUAT-1 in the rhizosphere of rice was stable. Interestingly, using denaturing gradient gel electrophoresis (PCR-DGGE), Kang et al. (2013) conducted a study on the persistence of *B. pumilus* WP8 in soil. The authors showed that the abundance of *B. pumilus* WP8 was stable up to 40 days in bulk soil. Besides, the strain changed the native soil bacterial community, particularly the dominant structure. However, these changes and persistence of this PGPB strain did not interfere with the efficiency of PGPB strain (Kang et al., 2013). Another study on the effects of *B. pumilus* on the indigenous microbiota was carried out using phospholipid fatty acid (PLFA) analysis (Probanza et al., 2001). Application of *B. pumilus* with *B. licheniformis* contributed to a change of the rhizosphere microbiota of stone pine (*Pinus pinea*), despite the low number of these strains at the end of the study. However, also in this case, these changes did not interfere with the efficiency of PGPB strain (Probanza et al., 2001). Moreover, Ramos et al. (2003) also documented the changes in the native microbiota of the rhizosphere [*Alnus glutinosa* (L.) Gaertn] after *B. pumilus* application using PLFA analysis (Ramos et al., 2003). Importantly, Win et al. (2018) assessed the response of the native microbiota to *B. pumilus* (strain TUAT-1) introduction using Next-Generation Sequencing (NGS); the study included microbiota of bulk soil, as well as rhizosphere and root endosphere of rice. For instance, *B. pumilus* application caused an increase in the population of Acidobacteriales and Desulfuromonadales and decreased the number of Xanthomonadales. Interestingly, the introduction of PGPB does not always result in alterations to the native bacterial community of soil or plant tissue. Dos Santos et al. (2022) did not observe the impact of *B. subtilis* application on the soybean microbial community.

Finally, it is worth adding that the mechanism of the impact of PGPB on the microbiota is still not fully understood and depends on a number of variables, e.g., chemical properties of soil, plant root exudates, plant development stage, and structure of indigenous microbiota of treated plants and soil type (Manfredini et al., 2021). In conclusion, it should also be noted that shifting the native microbiota in the right direction by PGPB may be one of the key factors in suppressing the phytopathogens (Chowdhury et al., 2015).

## 8. Conclusion

*Bacillus pumilus* is capable of producing lipopeptides, hydrolytic enzymes (e.g., chitinase and cellulase), and VOCs, and importantly is involved in triggering ISR. Thus, this species is a very good PGPB exhibiting a number of beneficial traits that are used to biocontrol a large number of fungal phytopathogens. However, there is still not a lot of studies using the strains on plants, particularly with the emphasis on studies in field conditions. Moreover, the mechanism of ISR elicitation by *B. pumilus* and other members of *Bacillus* spp. is still not fully elucidated. Based on the current knowledge, future research addressing this issue should focus specifically on the ISR induction by antibiotic lipopeptides, especially surfactin, which was detected in large quantities around the roots after the inoculation of *B. amyloliquefaciens*. Besides, due to the multitude of factors determining the composition of the native plant and soil microbiota, there is also a need for further research to determine the impact of *B. pumilus* on the native microbiota and its survival in bulk soil, rhizosphere, and plant tissues. Furthermore, studies profiling the transcriptome should be carried out in parallel, which would assess the RNA occurrence of key biocontrol agents in response to *B. pumilus* application. These studies should be conducted with recommended NGS sequencing techniques for the evaluation of microbiota after inoculation and qPCR for post-inoculation monitoring. This approach will allow a deeper insight into the relationship between the native microbiota and the inoculant and find some patterns which may have a direct impact on the efficiency of *B. pumilus* in biocontrol. Importantly, already commercialized strains should also be studied in order to make future formulations more effective and safe for the native microbiota.

## Author contributions

JD contributed to the conception of the review. JD, ZJ, IK, PK, and KK wrote the first draft of the manuscript. All authors contributed to manuscript revision, read, and approved the submitted version.
